# Supplementary figures and images for: Serotonin transporter genotype modulates resting state and predator stress-induced amygdala perfusion in mice in a sex-dependent manner
Source: PLoS One. 2021 Feb 19;16(2):e0247311. doi: 10.1371/journal.pone.0247311 (PMC7895400; doi:10.1371/journal.pone.0247311)

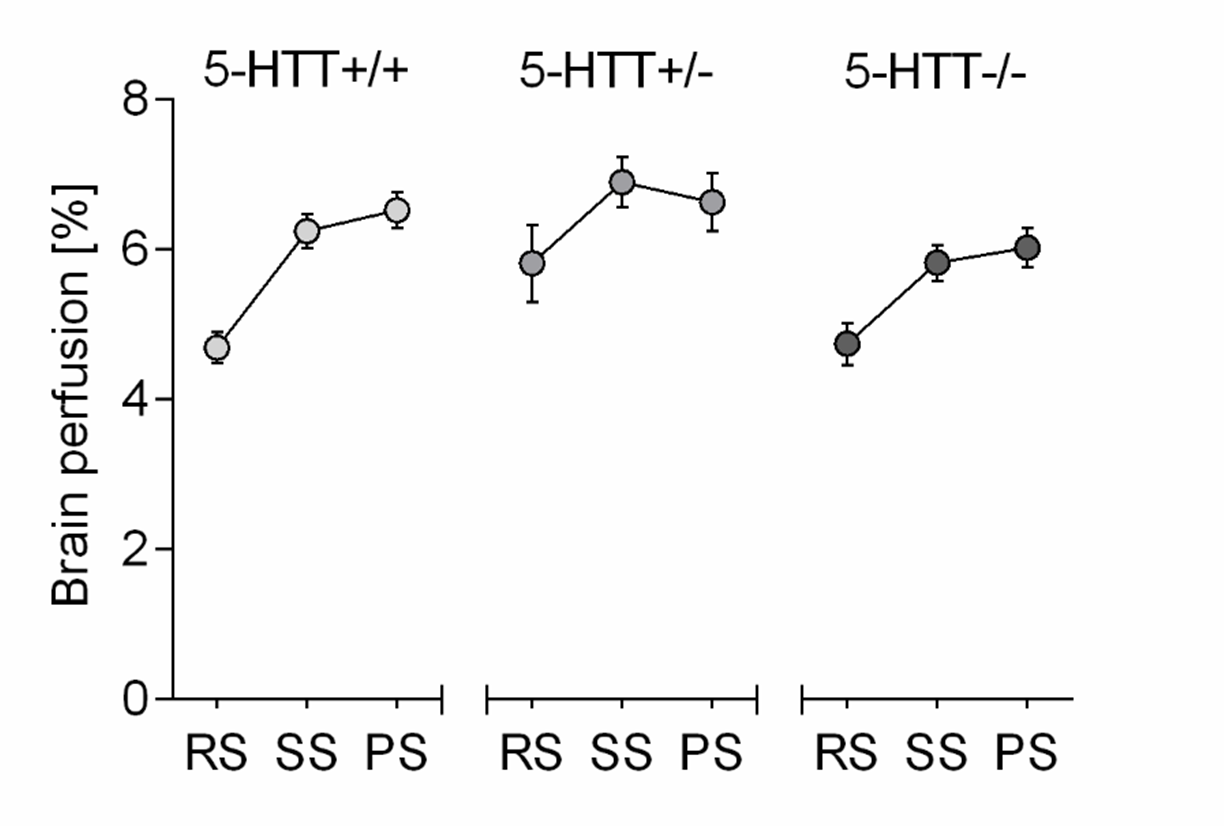

Supplement: S1 Fig — Cerebral perfusion levels during resting state (RS), stimulation state (SS) and post-stimulation state (PS) in whole brain of male mice of all three 5-Htt genotypes. Data represent mean perfusion level ± SEM. (TIF) [file pone.0247311.s001.tif]

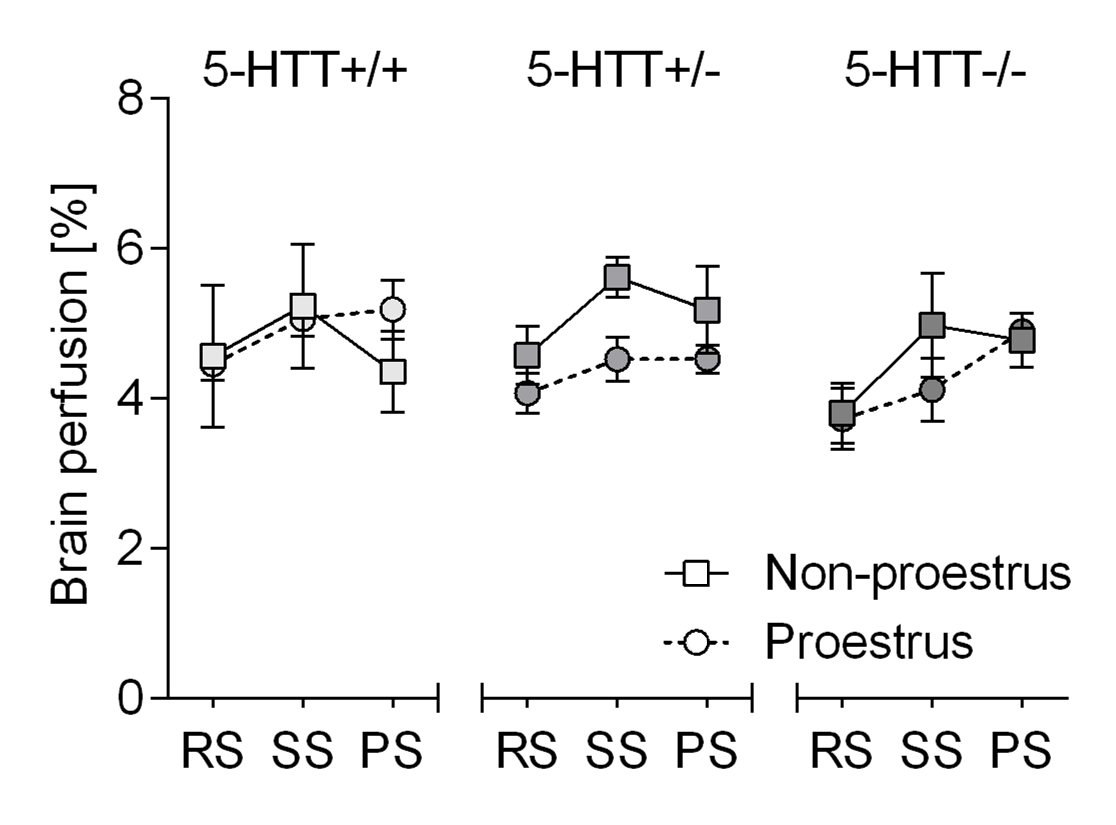

Supplement: S2 Fig — Cerebral perfusion levels during different fMRI states in whole brain of female mice of all three 5-Htt genotypes depending on their estrous stage receiving an aversive odor during SS. Data represent mean perfusion level ± SEM. (TIF) [file pone.0247311.s002.tif]

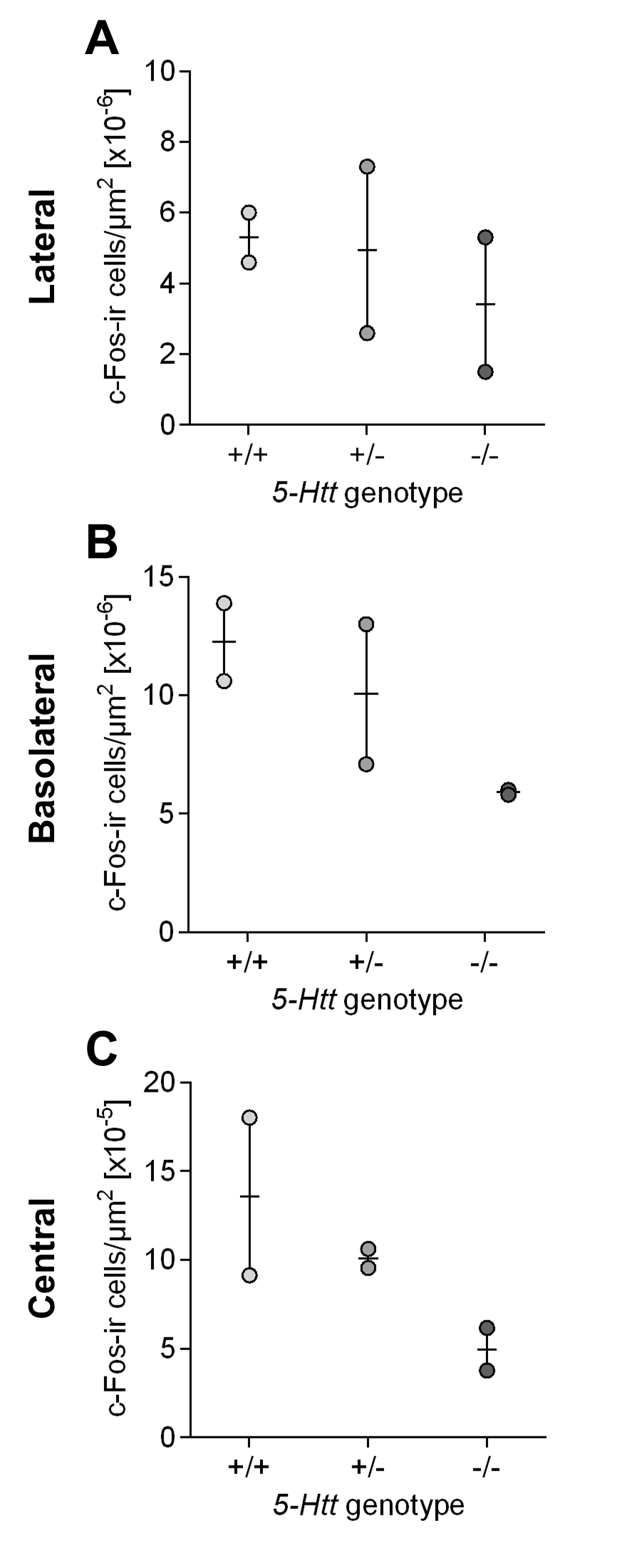

Supplement: S3 Fig — (A, B, C): Individual and mean density of c-Fos-immunoreactive cells (number of c-Fos-ir cells per μm2) in the lateral, basolateral and central amygdaloid nucleus of female mice with aversive rat odor exposure. (TIF) [file pone.0247311.s003.tif]
