# Supplementary material for: Serotonin transporter genotype modulates resting state and predator stress-induced amygdala perfusion in mice in a sex-dependent manner
Source: PLoS One. 2021 Feb 19;16(2):e0247311. doi: 10.1371/journal.pone.0247311 (PMC7895400; doi:10.1371/journal.pone.0247311)
Supplement: S1 Table — (DOCX) [file pone.0247311.s004.docx]

**S1 Table. Descriptive and inferential statistics of fMRI measurements and c-Fos immunohistochemistry with male mice of different *5-Htt* genotypes.**

1. Descriptive Statistics

|  | | 5-HTT+/+ | | | 5-HTT+/- | | | 5-HTT-/- | | |
| --- | --- | --- | --- | --- | --- | --- | --- | --- | --- | --- |
|  |  | n | Mean | SEM | n | Mean | SEM | n | Mean | SEM |
| Amygdala | RS | 8 | 3.317 | 0.166 | 9 | 4.825 | 0.391 | 12 | 3.933 | 0.264 |
|  | SS | 8 | 5.233 | 0.170 | 9 | 5.623 | 0.445 | 12 | 4.874 | 0.177 |
|  | PS | 8 | 5.832 | 0.336 | 9 | 5.837 | 0.459 | 12 | 5.261 | 0.284 |
|  | ΔSS/RS | 8 | 159.6 | 6.72 | 9 | 119.7 | 9.08 | 12 | 128.9 | 7.78 |
|  | ΔPS/RS | 8 | 177.0 | 9.18 | 9 | 122.7 | 6.85 | 12 | 139.7 | 10.80 |
|  | ΔPS/SS | 8 | 111.7 | 6.31 | 9 | 106.5 | 9.48 | 12 | 108.8 | 5.91 |
|  |  |  |  |  |  |  |  |  |  |  |
| Whole brain | RS | 8 | 4.690 | 0.205 | 9 | 5.817 | 0.512 | 12 | 4.746 | 0.280 |
|  | SS | 8 | 6.245 | 0.229 | 9 | 6.894 | 0.337 | 12 | 5.824 | 0.241 |
|  | PS | 8 | 6.525 | 0.243 | 9 | 6.630 | 0.384 | 12 | 6.026 | 0.261 |
|  | ΔSS/RS | 8 | 134.1 | 4.66 | 9 | 122.9 | 7.46 | 12 | 125.5 | 5.92 |
|  | ΔPS/RS | 8 | 140.0 | 4.97 | 9 | 117.0 | 6.26 | 12 | 130.2 | 7.14 |
|  | ΔPS/SS | 8 | 104.8 | 3.47 | 9 | 96.3 | 3.82 | 12 | 103.7 | 2.39 |
|  |  |  |  |  |  |  |  |  |  |  |
| c-Fos | LA | 4 | 1.96E-5 | 3.09E-6 | 4 | 7.66E-6 | 9.35E-7 | 6 | 7.16E-6 | 1.57E-6 |
|  | BLA | 4 | 2.05E-5 | 1.21E-6 | 4 | 7.67E-6 | 2.58E-6 | 6 | 6.84E-6 | 1.65E-6 |
|  | CeA | 4 | 3.23E-4 | 4.15E-5 | 4 | 1.62E-4 | 2.13E-5 | 6 | 1.56E-4 | 2.69E-5 |

2. Inferential Statistics

2.1. Testing for normality (Shapiro-Wilk test) and homogeneity of variances (Levene’s test)

|  | | Shapiro-Wilk test results | | | | | | | | |  | Levene’s test results | | | |
| --- | --- | --- | --- | --- | --- | --- | --- | --- | --- | --- | --- | --- | --- | --- | --- |
|  |  | 5-HTT+/+ | | | 5-HTT+/- | | | 5-HTT-/- | | |  |  | | | |
|  |  | df | W | *p* | df | W | *p* | df | W | *p* |  | df1 | df2 | F | *p* |
| Amygdala | RS | 8 | .948 | .696 | 9 | .882 | .164 | 12 | .857 | **.044** |  | 2 | 26 | 5.211 | **.012** |
|  | SS | 8 | .963 | .838 | 9 | .922 | .405 | 12 | .953 | .674 |  | 2 | 26 | 4.092 | **.029** |
|  | PS | 8 | .981 | .966 | 9 | .948 | .666 | 12 | .959 | .771 |  | 2 | 26 | 0.803 | .459 |
|  | ΔSS/RS | 8 | .975 | .936 | 9 | .947 | .653 | 12 | .982 | .992 |  | 2 | 26 | 0.509 | .607 |
|  | ΔPS/RS | 8 | .961 | .820 | 9 | .979 | .962 | 12 | .968 | .890 |  | 2 | 26 | 2.432 | .108 |
|  | ΔPS/SS | 8 | .912 | .367 | 9 | .834 | .049 | 12 | .944 | .546 |  | 2 | 26 | 0.259 | .774 |
|  |  |  |  |  |  |  |  |  |  |  |  |  |  |  |  |
| Whole brain | RS | 8 | .935 | .561 | 9 | .885 | .176 | 12 | .857 | **.045** |  | 2 | 26 | 5.318 | **.012** |
|  | SS | 8 | .861 | .122 | 9 | .899 | .246 | 12 | .959 | .767 |  | 2 | 26 | 0.195 | .824 |
|  | PS | 8 | .960 | .810 | 9 | .927 | .454 | 12 | .971 | .926 |  | 2 | 26 | 1.303 | .289 |
|  | ΔSS/RS | 8 | .937 | .579 | 9 | .946 | .644 | 12 | .942 | .522 |  | 2 | 26 | 1.134 | .337 |
|  | ΔPS/RS | 8 | .921 | .440 | 9 | .900 | .254 | 12 | .970 | .915 |  | 2 | 26 | 1.704 | .202 |
|  | ΔPS/SS | 8 | .902 | .303 | 9 | .945 | .639 | 12 | .975 | .954 |  | 2 | 26 | 0.222 | .802 |
|  |  |  |  |  |  |  |  |  |  |  |  |  |  |  |  |
| c-Fos | LA | 4 | .886 | .366 | 4 | .918 | .528 | 6 | .970 | .893 |  | 2 | 11 | 5.051 | **.028** |
|  | BLA | 4 | .941 | .658 | 4 | .899 | .428 | 6 | .964 | .850 |  | 2 | 11 | 0.897 | .436 |
|  | CeA | 4 | .905 | .458 | 4 | .845 | .210 | 6 | .927 | .553 |  | 2 | 11 | 0.716 | .510 |

2.2. Mauchly’s test of sphericity

| ROI | Within Subjects Effect | Mauchly’s W | Approx. Chi-Square | df | *p* |
| --- | --- | --- | --- | --- | --- |
| Amygdala | Phase | .974 | .647 | 2 | .724 |
| Brain | Phase | .821 | 4.929 | 2 | .085 |

2.3. ANOVA test results

|  | | Phase | | | | Phase x Genotype | | | | Genotype | | | |
| --- | --- | --- | --- | --- | --- | --- | --- | --- | --- | --- | --- | --- | --- |
|  |  | df1 | df2 | F | *p* | df1 | df2 | F | *p* | df1 | df2 | F | *p* |
| Amygdala | Perfusion | 2 | 52 | 35.63 | **.000** | 4 | 52 | 2.61 | **.046** | 2 | 26 | 2.68 | .087 |
|  | RS |  |  |  |  |  |  |  |  | 2 | 15.9 | 6.81 | **.007** |
|  | SS |  |  |  |  |  |  |  |  | 2 | 15.3 | 1.74 | .208 |
|  | PS |  |  |  |  |  |  |  |  | 2 | 26 | 0.94 | .405 |
|  | ΔSS/RS |  |  |  |  |  |  |  |  | 2 | 26 | 5.85 | **.008** |
|  | ΔPS/RS |  |  |  |  |  |  |  |  | 2 | 26 | 7.20 | **.003** |
|  | ΔPS/SS |  |  |  |  |  |  |  |  | 2 | 26 | 0.11 | .894 |
|  |  |  |  |  |  |  |  |  |  |  |  |  |  |
| Whole brain | Perfusion | 2 | 52 | 46.57 | **.000** | 4 | 52 | 1.86 | .131 | 2 | 26 | 3.03 | .066 |
|  | RS |  |  |  |  |  |  |  |  | 2 | 15.8 | 2.05 | .162 |
|  | SS |  |  |  |  |  |  |  |  | 2 | 26 | 4.08 | **.029** |
|  | PS |  |  |  |  |  |  |  |  | 2 | 26 | 1.26 | .302 |
|  | ΔSS/RS |  |  |  |  |  |  |  |  | 2 | 26 | 0.76 | .476 |
|  | ΔPS/RS |  |  |  |  |  |  |  |  | 2 | 26 | 2.73 | .084 |
|  | ΔPS/SS |  |  |  |  |  |  |  |  | 2 | 26 | 2.02 | .153 |
|  |  |  |  |  |  |  |  |  |  |  |  |  |  |
| c-Fos | LA |  |  |  |  |  |  |  |  | 2 | 6.1 | 6.53 | **.031** |
|  | BLA |  |  |  |  |  |  |  |  | 2 | 11 | 15.59 | **.001** |
|  | CeA |  |  |  |  |  |  |  |  | 2 | 11 | 8.88 | **.005** |

Welch’s ANOVA
