# Supplementary material for: Serotonin transporter genotype modulates resting state and predator stress-induced amygdala perfusion in mice in a sex-dependent manner
Source: PLoS One. 2021 Feb 19;16(2):e0247311. doi: 10.1371/journal.pone.0247311 (PMC7895400; doi:10.1371/journal.pone.0247311)
Supplement: S2 Table — (DOCX) [file pone.0247311.s005.docx]

**Table S1:** **Estrous cycle staging in female mice.**

| Estrous cycle stage | 5-HTT+/+ | 5-HTT+/- | 5-HTT-/- | Mice total [n] | Mice total [%] |
| --- | --- | --- | --- | --- | --- |
| Proestrus | 7 | 5 | 5 | 17 | 60.7 |
| Estrus | 2 | 2 | 1 | 5 | 17.9 |
| Metestrus | 0 | 0 | 1 | 1 | 3.6 |
| Diestrus | 0 | 2 | 3 | 5 | 17.9 |
| Mice total | 9 | 9 | 10 | 28 |  |

Additional control mice (neutral odor): 5-HTT+/+: estrus (*n*=1), 5-HTT+/-: metestrus (*n*=1), 5-HTT-/-: estrus (*n*=1), metestrus (*n*=1)
