# Supplementary material for: Serotonin transporter genotype modulates resting state and predator stress-induced amygdala perfusion in mice in a sex-dependent manner
Source: PLoS One. 2021 Feb 19;16(2):e0247311. doi: 10.1371/journal.pone.0247311 (PMC7895400; doi:10.1371/journal.pone.0247311)
Supplement: S3 Table — (DOCX) [file pone.0247311.s006.docx]

**S3 Table. Descriptive and inferential statistics (with data?) of fMRI measurements with female mice of different *5-Htt* genotypes.**

##### 1. Descriptive Statistics

|  | | Estrous  stage | 5-HTT+/+ | | | 5-HTT+/- | | | 5-HTT-/- | | |
| --- | --- | --- | --- | --- | --- | --- | --- | --- | --- | --- | --- |
|  |  |  | n | Mean | SEM | n | Mean | SEM | n | Mean | SEM |
| Amygdala | RS | proestrus | 7 | 3.042 | 0.186 | 5 | 3.042 | 0.161 | 5 | 2.855 | 0.341 |
|  |  | others | 2 | 2.798 | 0.735 | 4 | 3.446 | 0.519 | 5 | 2.831 | 0.319 |
|  |  | total | 9 | 2.988 | 0.191 | 9 | 3.222 | 0.239 | 10 | 2.843 | 0.220 |
|  | SS | proestrus | 7 | 4.056 | 0.274 | 5 | 3.747 | 0.332 | 5 | 3.226 | 0.296 |
|  |  | others | 2 | 4.312 | 0.701 | 4 | 4.885 | 0.571 | 5 | 4.471 | 0.498 |
|  |  | total | 9 | 4.113 | 0.243 | 9 | 4.252 | 0.353 | 10 | 3.849 | 0.343 |
|  | PS | proestrus | 7 | 4.043 | 0.338 | 5 | 3.490 | 0.165 | 5 | 4.044 | 0.084 |
|  |  | others | 2 | 3.202 | 0.464 | 4 | 4.310 | 0.478 | 5 | 3.835 | 0.328 |
|  |  | total | 9 | 3.856 | 0.296 | 9 | 3.855 | 0.258 | 10 | 3.940 | 0.163 |
|  | ΔSS/RS | proestrus | 7 | 134.3 | 7.95 | 5 | 124.6 | 14.10 | 5 | 116.0 | 9.36 |
|  |  | others | 2 | 158.5 | 16.56 | 4 | 150.0 | 22.29 | 5 | 160.3 | 13.66 |
|  |  | total | 9 | 139.6 | 7.56 | 9 | 135.9 | 12.57 | 10 | 138.1 | 10.74 |
|  | ΔPS/RS | proestrus | 7 | 134.6 | 11.24 | 5 | 115.7 | 6.59 | 5 | 151.9 | 22.00 |
|  |  | others | 2 | 118.3 | 14.48 | 4 | 135.5 | 26.86 | 5 | 139.2 | 12.83 |
|  |  | total | 9 | 131.0 | 9.24 | 9 | 124.5 | 12.02 | 10 | 145.6 | 12.19 |
|  | ΔPS/SS | proestrus | 7 | 101.7 | 9.29 | 5 | 95.9 | 8.77 | 5 | 130.0 | 12.78 |
|  |  | others | 2 | 74.5 | 1.35 | 4 | 89.3 | 7.95 | 5 | 87.9 | 6.97 |
|  |  | total | 9 | 95.7 | 8.15 | 9 | 92.9 | 5.76 | 10 | 108.9 | 9.81 |
|  |  |  |  |  |  |  |  |  |  |  |  |
| Whole-brain | RS | proestrus | 7 | 4.459 | 0.215 | 5 | 4.073 | 0.270 | 5 | 3.726 | 0.410 |
|  |  | others | 2 | 4.560 | 0.959 | 4 | 4.575 | 0.390 | 5 | 3.802 | 0.399 |
|  |  | total | 9 | 4.482 | 0.230 | 9 | 4.296 | 0.231 | 10 | 3.764 | 0.270 |
|  | SS | proestrus | 7 | 5.064 | 0.236 | 5 | 4.522 | 0.304 | 5 | 4.115 | 0.430 |
|  |  | others | 2 | 5.239 | 0.830 | 4 | 5.614 | 0.273 | 5 | 4.976 | 0.702 |
|  |  | total | 9 | 5.103 | 0.229 | 9 | 5.007 | 0.274 | 10 | 4.545 | 0.414 |
|  | PS | proestrus | 7 | 5.189 | 0.396 | 5 | 4.528 | 0.192 | 5 | 4.895 | 0.108 |
|  |  | others | 2 | 4.354 | 0.541 | 4 | 5.186 | 0.581 | 5 | 4.774 | 0.362 |
|  |  | total | 9 | 5.004 | 0.339 | 9 | 4.820 | 0.283 | 10 | 4.834 | 0.179 |
|  | ΔSS/RS | proestrus | 7 | 114.3 | 5.25 | 5 | 112.4 | 9.59 | 5 | 112.2 | 7.49 |
|  |  | others | 2 | 116.2 | 6.23 | 4 | 124.5 | 8.78 | 5 | 130.5 | 10.91 |
|  |  | total | 9 | 114.7 | 4.15 | 9 | 117.8 | 6.55 | 10 | 121.3 | 6.95 |
|  | ΔPS/RS | proestrus | 7 | 116.4 | 5.84 | 5 | 113.0 | 8.33 | 5 | 139.3 | 18.04 |
|  |  | others | 2 | 97.3 | 8.60 | 4 | 113.4 | 7.61 | 5 | 128.8 | 10.80 |
|  |  | total | 9 | 112.1 | 5.46 | 9 | 113.2 | 5.38 | 10 | 134.1 | 10.07 |
|  | ΔPS/SS | proestrus | 7 | 102.5 | 5.45 | 5 | 101.9 | 7.86 | 5 | 124.5 | 13.55 |
|  |  | others | 2 | 83.6 | 2.92 | 4 | 91.9 | 7.22 | 5 | 100.1 | 8.14 |
|  |  | total | 9 | 98.3 | 5.03 | 9 | 97.4 | 5.38 | 10 | 112.3 | 8.49 |

##### 2. Inferential Statistics

2.1. Testing for normality (Shapiro-Wilk test) and homogeneity of variances (Levene’s test)

2.1.1. Shapiro-Wilk tests

2.1.1.1. Factor Genotype

|  | | Shapiro-Wilk test results | | | | | | | | |
| --- | --- | --- | --- | --- | --- | --- | --- | --- | --- | --- |
|  |  | 5-HTT+/+ | | | 5-HTT+/- | | | 5-HTT-/- | | |
|  |  | df | W | *p* | df | W | *p* | df | W | *p* |
| Amygdala | RS | 9 | .980 | .966 | 9 | .976 | .942 | 10 | .968 | .870 |
|  | SS | 9 | .971 | .903 | 9 | .931 | .494 | 10 | .892 | .180 |
|  | PS | 9 | .859 | .094 | 9 | .895 | .223 | 10 | .930 | .446 |
|  | ΔSS/RS | 9 | .940 | .580 | 9 | .773 | .010 | 10 | .937 | .516 |
|  | ΔPS/RS | 9 | .920 | .396 | 9 | .789 | .015 | 10 | .850 | .058 |
|  | ΔPS/SS | 9 | .898 | .240 | 9 | .914 | .347 | 10 | .937 | .517 |
|  |  |  |  |  |  |  |  |  |  |  |
| Whole brain | RS | 9 | .958 | .780 | 9 | .886 | .181 | 10 | .941 | .569 |
|  | SS | 9 | .879 | .153 | 9 | .954 | .732 | 10 | .916 | .325 |
|  | PS | 9 | .784 | .014 | 9 | .775 | .011 | 10 | .967 | .859 |
|  | ΔSS/RS | 9 | .825 | .039 | 9 | .778 | .011 | 10 | .967 | .865 |
|  | ΔPS/RS | 9 | .969 | .888 | 9 | .852 | .078 | 10 | .840 | **.044** |
|  | ΔPS/SS | 9 | .873 | .131 | 9 | .836 | .052 | 10 | .878 | .123 |

2.1.1.2. Factor Estrous stage

|  | | Shapiro Wilk test results | | | | | |
| --- | --- | --- | --- | --- | --- | --- | --- |
|  |  | Proestrus | | | Others (Non-proestrus) | | |
|  |  | df | W | *p* | df | W | *p* |
| Amygdala | RS | 17 | .977 | .920 | 11 | .906 | .221 |
|  | SS | 17 | .982 | .976 | 11 | .882 | .112 |
|  | PS | 17 | .816 | **.003** | 11 | .934 | .450 |
|  | ΔSS/RS | 17 | .860 | **.015** | 11 | .945 | .578 |
|  | ΔPS/RS | 17 | .862 | **.017** | 11 | .887 | .127 |
|  | ΔPS/SS | 17 | .934 | .251 | 11 | .911 | .248 |
|  |  |  |  |  |  |  |  |
| Whole brain | RS | 17 | .927 | .195 | 11 | .955 | .711 |
|  | SS | 17 | .954 | .528 | 11 | .950 | .650 |
|  | PS | 17 | .714 | **.000** | 11 | .899 | .180 |
|  | ΔSS/RS | 17 | .863 | **.017** | 11 | .928 | .395 |
|  | ΔPS/RS | 17 | .814 | **.003** | 11 | .933 | .437 |
|  | ΔPS/SS | 17 | .875 | **.027** | 11 | .773 | **.004** |

2.1.2. Homogeneity of variance

|  | | Levene’s test results | | | |
| --- | --- | --- | --- | --- | --- |
|  |  | df1 | df2 | F | *p* |
| Amygdala | RS | 5 | 22 | 1.384 | .269 |
|  | SS | 5 | 22 | 1.152 | .363 |
|  | PS | 5 | 22 | 1.178 | .351 |
|  | ΔSS/RS | 5 | 22 | 1.368 | .274 |
|  | ΔPS/RS | 5 | 22 | 1.465 | .241 |
|  | ΔPS/SS | 5 | 22 | 2.151 | .097 |
|  |  |  |  |  |  |
| Whole brain | RS | 5 | 22 | 0.822 | .547 |
|  | SS | 5 | 22 | 3.158 | **.027** |
|  | PS | 5 | 22 | 1.204 | .340 |
|  | ΔSS/RS | 5 | 22 | 0.817 | .551 |
|  | ΔPS/RS | 5 | 22 | 1.498 | .231 |
|  | ΔPS/SS | 5 | 22 | 5.956 | **.001** |

2.2. Mauchly’s test of sphericity

| ROI | Within Subjects Effect | Mauchly’s W | Approx. Chi-Square | df | *p* |
| --- | --- | --- | --- | --- | --- |
| Amygdala | Phase | .980 | .429 | 2 | .807 |
| Brain | Phase | .941 | 1.282 | 2 | .527 |

2.3 ANOVA test results

2.3.1 Three-way mixed ANOVA

|  | Amygdala perfusion | | | | Whole brain perfusion | | | |
| --- | --- | --- | --- | --- | --- | --- | --- | --- |
|  | df1 | df2 | F | *p* | df1 | df2 | F | *p* |
| Phase | 2 | 44 | 26.39 | **.000** | 2 | 44 | 13.68 | **.000** |
| Phase x Genotype | 4 | 44 | 0.92 | .462 | 4 | 44 | 1.53 | .211 |
| Phase x Estrous | 2 | 44 | 5.37 | **.009** | 2 | 44 | 3.70 | **.033** |
| Phase x Genotype x Estrous | 4 | 44 | 0.87 | .487 | 4 | 44 | 0.63 | .642 |
| Genotype | 2 | 22 | 0.53 | .594 | 2 | 22 | 0.85 | .439 |
| Estrous | 1 | 22 | 1.27 | .272 | 1 | 22 | 0.85 | .368 |
| Genotype x Estrous | 2 | 22 | 1.36 | .276 | 2 | 22 | 0.74 | .490 |

2.3.2 Two-way ANOVAs

|  | | Genotype | | | | Estrous | | | | Genotype x Estrous | | | |
| --- | --- | --- | --- | --- | --- | --- | --- | --- | --- | --- | --- | --- | --- |
|  |  | df1 | df2 | F | *p* | df1 | df2 | F | *p* | df1 | df2 | F | *p* |
| Amygdala | RS | 2 | 22 | 0.84 | .444 | 1 | 22 | 0.02 | .876 | 2 | 22 | 0.44 | .651 |
|  | SS | 2 | 22 | 0.71 | .505 | 1 | 22 | 6.04 | **.022** | 2 | 22 | 0.68 | .518 |
|  | PS | 2 | 22 | 0.43 | .654 | 1 | 22 | 0.07 | .791 | 2 | 22 | 2.75 | .086 |
|  | ΔSS/RS | 2 | 22 | 0.21 | .814 | 1 | 22 | 6.98 | **.015** | 2 | 22 | 0.33 | .722 |
|  | ΔPS/RS | 2 | 22 | 0.91 | .418 | 1 | 22 | 0.04 | .835 | 2 | 22 | 1.61 | .222 |
|  | ΔPS/SS | 2 | 22 | 2.24 | .131 | 1 | 22 | 8.36 | **.008** | 2 | 22 | 0.65 | .533 |
|  |  |  |  |  |  |  |  |  |  |  |  |  |  |
| Whole brain | RS | 2 | 22 | 2.07 | .150 | 1 | 22 | 0.50 | .487 | 2 | 22 | 0.20 | .818 |
|  | SS | 2 | 22 | 1.04 | .370 | 1 | 22 | 3.34 | .081 | 2 | 22 | 0.44 | .650 |
|  | PS | 2 | 22 | 0.02 | .980 | 1 | 22 | 0.09 | .768 | 2 | 22 | 1.55 | .235 |
|  | ΔSS/RS | 2 | 22 | 0.21 | .812 | 1 | 22 | 2.06 | .165 | 2 | 22 | 0.38 | .686 |
|  | ΔPS/RS | 2 | 22 | 3.05 | .068 | 1 | 22 | 1.00 | .327 | 2 | 22 | 0.32 | .731 |
|  | ΔPS/SS | 2 | 22 | 2.44 | .111 | 1 | 22 | 5.15 | **.033** | 2 | 22 | 0.33 | .721 |
